# (2*E*)-Methyl 2-(7-benz­yloxy-1-naphth­yl)-3-meth­oxy­acrylate

**DOI:** 10.1107/S1600536810020994

**Published:** 2010-06-09

**Authors:** Xin Zhu, Heping Li, Jing Yang, Hui Li

**Affiliations:** a College of Pharmacy, Henan University of Traditional Chinese Medicine, Zhengzhou, Henan 450008, People’s Republic of China; b Department of Chemistry, Zhengzhou University, Zhengzhou, Henan 450052, People’s Republic of China

## Abstract

In the title compound, C_22_H_20_O_4_, the dihedral angle between the phenyl and naphthalene ring systems is 86.10 (10)°.  The methoxyacrylate group is disordered over two orientations in a 0.905 (3):0.095 (3) ratio.

## Related literature

For bond-length data, see: Allen *et al.* (1987 ▶). For a related synthesis and crystal structure, see: Fun *et al.* (2008 ▶). For general background to and applications of compounds containing aromatic rings, see: Gunatilaka (2006 ▶); Kozikowski *et al.* (2000 ▶). Methyl­ene carbonyl compounds are often found in biologically active natural compounds, see: Gotthardt & Weisshuhn (1978 ▶); Shono *et al.* (1979 ▶).
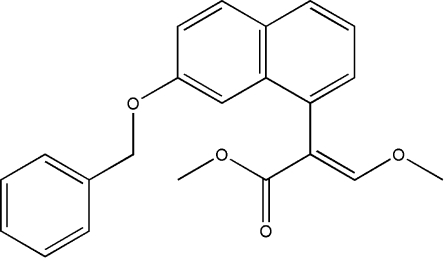


## Experimental

### 

#### Crystal data

C_22_H_20_O_4_
*M* *_r_* = 348.38
Monoclinic, 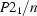

*a* = 10.996 (5) Å
*b* = 7.873 (5) Å
*c* = 21.417 (5) Å
β = 102.493 (5)°
*V* = 1810.2 (15) Å^3^
*Z* = 4
Mo *K*α radiation
μ = 0.09 mm^−1^
*T* = 293 K
0.4 × 0.4 × 0.3 mm

#### Data collection

Bruker SMART CCD area-detector diffractometer
Absorption correction: multi-scan (*SADABS*; Bruker, 2000 ▶) *T*_min_ = 0.826, *T*_max_ = 0.973
21877 measured reflections
4203 independent reflections
3273 reflections with *I* > 2σ(*I*)
*R*_int_ = 0.025

#### Refinement

*R*[*F* ^2^ > 2σ(*F* ^2^)] = 0.044
*wR*(*F* ^2^) = 0.131
*S* = 1.04
4203 reflections
247 parameters
H-atom parameters constrained
Δρ_max_ = 0.19 e Å^−3^
Δρ_min_ = −0.17 e Å^−3^

### 

Data collection: *SMART* (Bruker, 2001 ▶); cell refinement: *SAINT* (Bruker, 1999 ▶); data reduction: *SAINT*; program(s) used to solve structure: *SHELXS97* (Sheldrick, 2008 ▶); program(s) used to refine structure: *SHELXL97* (Sheldrick, 2008 ▶); molecular graphics: *SHELXTL* (Sheldrick, 2008 ▶); software used to prepare material for publication: *SHELXL97*.

## Supplementary Material

Crystal structure: contains datablocks I, global. DOI: 10.1107/S1600536810020994/zq2038sup1.cif

Structure factors: contains datablocks I. DOI: 10.1107/S1600536810020994/zq2038Isup2.hkl

Additional supplementary materials:  crystallographic information; 3D view; checkCIF report

## Figures and Tables

**Fig. 1.**
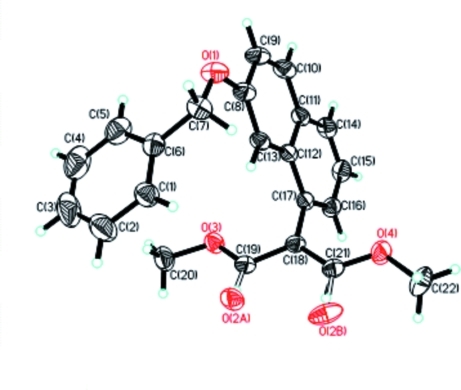
The molecular structure of (I) showing 30% probability displacement ellipsoids and the atomic numbering.

## supplementary crystallographic information

### Comment

Ether compounds containing aromatic rings are present in many bioactivity compounds and are useful intermediates in organic synthesis (Kozikowski *et al.*, 2000; Gunatilaka, 2006). Methylene carbonyl compounds have been studied extensively in recent years, since they are not only useful as synthetic intermediates but also often found in biologically active natural compounds (Gotthardt *et al.*, 1978; Shono *et al.*, 1979). Here we report the synthesis and the crystal structure of the title compound C_22_H_20_O_4_, namely (*E*)-methyl 2-(7-(benzyloxy)naphthalen-1-yl)-3-methoxyacrylate (I).

In the crystal structure, the molecule of (I) adopts two different conformations according to the relative position of the methoxyacrylate group. The minor and major conformers could be distinguished by the disorder of the oxygen atom O2 of the carbonyl group which occupies two different sites A (bound to C19) and B (bound to C21), the refined site-occupancy factors are 0.905 (3) and 0.095 (3), respectively. The naphthalene and benzene rings are essentially planar with r.m.s. deviations of 0.0036 (14) Å and 0.0001 (3) Å, respectively. The phenyl ring is almost perpendicular to the naphthalene ring, the dihedral angle between them is 86.10 (10)°, which is different from that in methyl 2-(7-benzyloxy-1-naphthyl)-2-oxoacetate, of 43.78 (7)° (Fun *et al.*, 2008). The dihedral angle between the mean plane of the methoxyvinyl and methoxycarbonyl groups is only 6.47 (18)°. The latter long and almost planar substituent plays an important role in the whole folded conformation of (I). Nevertheless, no classic hydrogen bonds were found in the crystal structure.

### Experimental

Methyl 2-(7-benzyloxy-1-naphthyl)-2-oxoacetate was synthesized according with a published procedure (Fun *et al.*, 2008). To a solution of methyl 2-(7-benzyloxy-1-naphthyl)-2-oxoacetate (4 g, 12.5 mmol) in dry THF (10 ml) was slowly added an excess of (methoxymethyl)triphenylphosphonium chloride (5.1 g, 20 mmol) in dry ether and 2.5 *M* butyl lithium in hexane (6 ml) under nitrogen. The mixture was allowed to warm to room temperature for 24 h, and then was quenched with dilute hydrochloric acid. The crude product was purified by column chromatography with petroleum ether - ethyl acetate (5: 1) as the eluent, to afford I as a white powder (0.5 g, 11.9%). Single crystals suitable for a X-ray analysis were obtained by slow evaporation from ethanol at room temperature for several days (m.p. 125–126°C).

### Refinement

All H atoms were positioned geometrically and refined as riding atoms, with C—H = 0.93Å and *U*_iso _= 1.2U_eq_(C) for aromatic hydrogen atoms, with C—H = 0.97 Å and *U*_iso _= 1.2U_eq_(C) for methylene hydrogen atoms, and with C—H=0.96 Å and *U*_iso _= 1.5U_eq_(C) for methyl hydrogen atoms. The minor and major conformers could be distinguished by the disorder of the oxygen atom O2 of the carbonyl group which occupies two different sites A (bound to C19) and B (bound to C21), the refined site-occupancy factors are 0.905 (3) and 0.095 (3), respectively.

### Crystal data

**Table:**

C_22_H_20_O_4_	*F*(000) = 736
*M**_r_* = 348.38	*D*_x_ = 1.278 Mg m^−^^3^
Monoclinic, *P*2_1_/*n*	Melting point = 398–399 K
Hall symbol: -P 2yn	Mo *K*α radiation, λ = 0.71073 Å
*a* = 10.996 (5) Å	Cell parameters from 7659 reflections
*b* = 7.873 (5) Å	θ = 2.3–27.6°
*c* = 21.417 (5) Å	µ = 0.09 mm^−^^1^
β = 102.493 (5)°	*T* = 293 K
*V* = 1810.2 (15) Å^3^	Block, colorless
*Z* = 4	0.4 × 0.4 × 0.3 mm

### Data collection

**Table:**

Bruker SMART CCD area-detector diffractometer	4203 independent reflections
Radiation source: fine-focus sealed tube	3273 reflections with *I* > 2σ(*I*)
graphite	*R*_int_ = 0.025
phi and ω scans	θ_max_ = 27.6°, θ_min_ = 2.3°
Absorption correction: multi-scan (*SADABS*; Bruker, 2000)	*h* = −14→14
*T*_min_ = 0.826, *T*_max_ = 0.973	*k* = −10→9
21877 measured reflections	*l* = −27→27

### Refinement

**Table:**

Refinement on *F*^2^	Primary atom site location: structure-invariant direct methods
Least-squares matrix: full	Secondary atom site location: difference Fourier map
*R*[*F*^2^ > 2σ(*F*^2^)] = 0.044	Hydrogen site location: inferred from neighbouring sites
*wR*(*F*^2^) = 0.131	H-atom parameters constrained
*S* = 1.04	*w* = 1/[σ^2^(*F*_o_^2^) + (0.0575*P*)^2^ + 0.3948*P*] where *P* = (*F*_o_^2^ + 2*F*_c_^2^)/3
4203 reflections	(Δ/σ)_max_ = 0.020
247 parameters	Δρ_max_ = 0.19 e Å^−^^3^
0 restraints	Δρ_min_ = −0.17 e Å^−^^3^

### Special details

**Table:**

Geometry. All e.s.d.'s (except the e.s.d. in the dihedral angle between two l.s. planes) are estimated using the full covariance matrix. The cell e.s.d.'s are taken into account individually in the estimation of e.s.d.'s in distances, angles and torsion angles; correlations between e.s.d.'s in cell parameters are only used when they are defined by crystal symmetry. An approximate (isotropic) treatment of cell e.s.d.'s is used for estimating e.s.d.'s involving l.s. planes.
Refinement. Refinement of *F*^2^ against ALL reflections. The weighted *R*-factor *wR* and goodness of fit *S* are based on *F*^2^, conventional *R*-factors *R* are based on *F*, with *F* set to zero for negative *F*^2^. The threshold expression of *F*^2^ > σ(*F*^2^) is used only for calculating *R*-factors(gt) *etc*. and is not relevant to the choice of reflections for refinement. *R*-factors based on *F*^2^ are statistically about twice as large as those based on *F*, and *R*-factors based on ALL data will be even larger.

### Fractional atomic coordinates and isotropic or equivalent isotropic displacement parameters (Å^2^)

**Table:**

	*x*	*y*	*z*	*U*_iso_*/*U*_eq_	Occ. (<1)
C1	0.89564 (15)	0.8419 (2)	0.43935 (8)	0.0617 (4)	
H1	0.9075	0.9125	0.4750	0.074*	
C2	0.9022 (2)	0.6687 (3)	0.44746 (13)	0.0899 (7)	
H2	0.9175	0.6215	0.4882	0.108*	
C3	0.8857 (2)	0.5660 (3)	0.39409 (18)	0.1101 (9)	
H3	0.8900	0.4486	0.3991	0.132*	
C4	0.8631 (2)	0.6339 (3)	0.33425 (16)	0.1042 (8)	
H4	0.8528	0.5629	0.2988	0.125*	
C5	0.85536 (18)	0.8070 (3)	0.32603 (10)	0.0768 (5)	
H5	0.8392	0.8531	0.2851	0.092*	
C6	0.87165 (13)	0.91229 (19)	0.37869 (7)	0.0515 (3)	
C7	0.85480 (15)	1.1015 (2)	0.37234 (7)	0.0554 (4)	
H7A	0.7704	1.1295	0.3755	0.067*	
H7B	0.9111	1.1558	0.4079	0.067*	
C8	0.99755 (14)	1.19352 (19)	0.30829 (6)	0.0497 (3)	
C9	1.00816 (16)	1.2723 (2)	0.25038 (7)	0.0591 (4)	
H9	0.9368	1.3001	0.2200	0.071*	
C10	1.12174 (16)	1.3073 (2)	0.23908 (7)	0.0573 (4)	
H10	1.1274	1.3592	0.2008	0.069*	
C11	1.23257 (14)	1.26688 (17)	0.28418 (6)	0.0464 (3)	
C12	1.22164 (13)	1.18630 (16)	0.34233 (6)	0.0411 (3)	
C13	1.10149 (13)	1.15120 (17)	0.35316 (6)	0.0437 (3)	
H13	1.0934	1.0991	0.3910	0.052*	
C14	1.35110 (16)	1.3059 (2)	0.27324 (7)	0.0563 (4)	
H14	1.3578	1.3577	0.2351	0.068*	
C15	1.45600 (16)	1.2686 (2)	0.31784 (7)	0.0580 (4)	
H15	1.5338	1.2961	0.3103	0.070*	
C16	1.44661 (14)	1.18867 (19)	0.37519 (7)	0.0513 (3)	
H16	1.5189	1.1635	0.4053	0.062*	
C17	1.33331 (13)	1.14669 (16)	0.38795 (6)	0.0421 (3)	
C18	1.32816 (12)	1.06687 (17)	0.45059 (6)	0.0431 (3)	
C19	1.28723 (12)	0.89191 (18)	0.45565 (6)	0.0457 (3)	
H19	1.2753	0.8435	0.4935	0.055*	0.095 (3)
O2A	1.26989 (13)	0.82617 (16)	0.50464 (5)	0.0647 (4)	0.905 (3)
C20	1.22339 (19)	0.6374 (2)	0.39907 (9)	0.0704 (5)	
H20A	1.2762	0.5730	0.4323	0.106*	
H20B	1.2236	0.5868	0.3583	0.106*	
H20C	1.1400	0.6380	0.4060	0.106*	
C21	1.36400 (13)	1.15235 (19)	0.50605 (6)	0.0495 (3)	
H21	1.3621	1.0975	0.5443	0.059*	0.905 (3)
O2B	1.3729 (13)	1.076 (2)	0.5625 (5)	0.084 (5)	0.095 (3)
C22	1.43189 (19)	1.3923 (3)	0.56949 (8)	0.0780 (5)	
H22A	1.3574	1.4053	0.5855	0.117*	
H22B	1.4680	1.5019	0.5659	0.117*	
H22C	1.4902	1.3228	0.5984	0.117*	
O1	0.87669 (10)	1.16997 (16)	0.31413 (5)	0.0632 (3)	
O3	1.26886 (10)	0.80927 (12)	0.39999 (5)	0.0546 (3)	
O4	1.40199 (12)	1.31265 (15)	0.50759 (5)	0.0662 (3)	

### Atomic displacement parameters (Å^2^)

**Table:**

	*U*^11^	*U*^22^	*U*^33^	*U*^12^	*U*^13^	*U*^23^
C1	0.0605 (9)	0.0539 (9)	0.0707 (10)	0.0037 (7)	0.0145 (8)	0.0037 (8)
C2	0.0809 (13)	0.0625 (12)	0.1244 (19)	0.0085 (10)	0.0181 (12)	0.0259 (12)
C3	0.0878 (16)	0.0439 (11)	0.194 (3)	0.0031 (10)	0.0207 (18)	−0.0089 (16)
C4	0.0886 (15)	0.0765 (15)	0.140 (2)	0.0046 (12)	0.0081 (15)	−0.0512 (16)
C5	0.0719 (11)	0.0777 (13)	0.0778 (12)	0.0062 (9)	0.0099 (9)	−0.0239 (10)
C6	0.0437 (7)	0.0521 (8)	0.0597 (8)	0.0019 (6)	0.0135 (6)	−0.0058 (7)
C7	0.0574 (8)	0.0559 (9)	0.0572 (8)	0.0078 (7)	0.0216 (7)	0.0049 (7)
C8	0.0570 (8)	0.0514 (8)	0.0415 (7)	0.0089 (6)	0.0122 (6)	0.0052 (6)
C9	0.0684 (10)	0.0673 (10)	0.0396 (7)	0.0144 (8)	0.0074 (7)	0.0125 (7)
C10	0.0792 (11)	0.0581 (9)	0.0362 (7)	0.0076 (8)	0.0160 (7)	0.0133 (6)
C11	0.0671 (9)	0.0404 (7)	0.0332 (6)	−0.0003 (6)	0.0144 (6)	0.0016 (5)
C12	0.0587 (8)	0.0342 (6)	0.0314 (6)	0.0007 (5)	0.0118 (5)	−0.0012 (5)
C13	0.0580 (8)	0.0413 (7)	0.0332 (6)	0.0042 (6)	0.0129 (5)	0.0055 (5)
C14	0.0783 (10)	0.0531 (9)	0.0423 (7)	−0.0088 (7)	0.0238 (7)	0.0048 (6)
C15	0.0643 (9)	0.0628 (10)	0.0515 (8)	−0.0155 (8)	0.0223 (7)	−0.0018 (7)
C16	0.0568 (8)	0.0534 (8)	0.0438 (7)	−0.0064 (7)	0.0111 (6)	−0.0024 (6)
C17	0.0571 (8)	0.0377 (6)	0.0326 (6)	−0.0025 (6)	0.0119 (5)	−0.0024 (5)
C18	0.0478 (7)	0.0468 (7)	0.0341 (6)	0.0029 (6)	0.0079 (5)	0.0027 (5)
C19	0.0497 (7)	0.0477 (8)	0.0396 (6)	0.0056 (6)	0.0092 (5)	0.0053 (6)
O2A	0.0923 (10)	0.0599 (8)	0.0444 (7)	−0.0086 (6)	0.0204 (6)	0.0093 (5)
C20	0.0862 (12)	0.0485 (9)	0.0765 (11)	−0.0104 (8)	0.0171 (9)	−0.0040 (8)
C21	0.0541 (8)	0.0563 (9)	0.0373 (6)	0.0015 (7)	0.0080 (6)	0.0019 (6)
O2B	0.084 (9)	0.134 (14)	0.030 (6)	0.019 (9)	0.008 (5)	0.004 (7)
C22	0.0847 (12)	0.0914 (14)	0.0562 (10)	−0.0192 (10)	0.0114 (8)	−0.0297 (9)
O1	0.0549 (6)	0.0787 (8)	0.0559 (6)	0.0109 (5)	0.0117 (5)	0.0176 (5)
O3	0.0762 (7)	0.0432 (5)	0.0462 (5)	−0.0023 (5)	0.0168 (5)	−0.0016 (4)
O4	0.0954 (9)	0.0593 (7)	0.0424 (6)	−0.0142 (6)	0.0114 (5)	−0.0093 (5)

### Geometric parameters (Å, °)

**Table:**

C1—C2	1.375 (3)	C12—C13	1.417 (2)
C1—C6	1.384 (2)	C12—C17	1.4285 (19)
C1—H1	0.9300	C13—H13	0.9300
C2—C3	1.380 (4)	C14—C15	1.361 (2)
C2—H2	0.9300	C14—H14	0.9300
C3—C4	1.361 (4)	C15—C16	1.404 (2)
C3—H3	0.9300	C15—H15	0.9300
C4—C5	1.375 (3)	C16—C17	1.372 (2)
C4—H4	0.9300	C16—H16	0.9300
C5—C6	1.380 (2)	C17—C18	1.4936 (17)
C5—H5	0.9300	C18—C21	1.3477 (19)
C6—C7	1.504 (2)	C18—C19	1.460 (2)
C7—O1	1.4255 (17)	C19—O3	1.3345 (16)
C7—H7A	0.9700	C19—H19	0.9300
C7—H7B	0.9700	C20—O3	1.441 (2)
C8—C13	1.366 (2)	C20—H20A	0.9600
C8—O1	1.3741 (19)	C20—H20B	0.9600
C8—C9	1.414 (2)	C20—H20C	0.9600
C9—C10	1.351 (2)	C21—O4	1.328 (2)
C9—H9	0.9300	C21—H21	0.9300
C10—C11	1.418 (2)	C22—O4	1.4388 (18)
C10—H10	0.9300	C22—H22A	0.9600
C11—C14	1.407 (2)	C22—H22B	0.9600
C11—C12	1.4258 (17)	C22—H22C	0.9600
			
C2—C1—C6	120.64 (19)	C8—C13—C12	120.35 (12)
C2—C1—H1	119.7	C8—C13—H13	119.8
C6—C1—H1	119.7	C12—C13—H13	119.8
C1—C2—C3	118.9 (2)	C15—C14—C11	120.73 (13)
C1—C2—H2	120.6	C15—C14—H14	119.6
C3—C2—H2	120.6	C11—C14—H14	119.6
C4—C3—C2	121.0 (2)	C14—C15—C16	119.95 (14)
C4—C3—H3	119.5	C14—C15—H15	120.0
C2—C3—H3	119.5	C16—C15—H15	120.0
C3—C4—C5	120.2 (2)	C17—C16—C15	121.60 (14)
C3—C4—H4	119.9	C17—C16—H16	119.2
C5—C4—H4	119.9	C15—C16—H16	119.2
C4—C5—C6	119.8 (2)	C16—C17—C12	119.62 (12)
C4—C5—H5	120.1	C16—C17—C18	119.51 (12)
C6—C5—H5	120.1	C12—C17—C18	120.81 (11)
C5—C6—C1	119.45 (17)	C21—C18—C19	116.21 (12)
C5—C6—C7	121.96 (16)	C21—C18—C17	121.38 (13)
C1—C6—C7	118.43 (14)	C19—C18—C17	122.39 (11)
O1—C7—C6	114.39 (13)	O3—C19—C18	112.53 (11)
O1—C7—H7A	108.7	O3—C19—H19	123.7
C6—C7—H7A	108.7	C18—C19—H19	123.7
O1—C7—H7B	108.7	O3—C20—H20A	109.5
C6—C7—H7B	108.7	O3—C20—H20B	109.5
H7A—C7—H7B	107.6	H20A—C20—H20B	109.5
C13—C8—O1	125.56 (12)	O3—C20—H20C	109.5
C13—C8—C9	120.59 (14)	H20A—C20—H20C	109.5
O1—C8—C9	113.83 (13)	H20B—C20—H20C	109.5
C10—C9—C8	120.10 (14)	O4—C21—C18	121.81 (13)
C10—C9—H9	120.0	O4—C21—H21	119.1
C8—C9—H9	120.0	C18—C21—H21	119.1
C9—C10—C11	121.59 (13)	O4—C22—H22A	109.5
C9—C10—H10	119.2	O4—C22—H22B	109.5
C11—C10—H10	119.2	H22A—C22—H22B	109.5
C14—C11—C10	121.88 (13)	O4—C22—H22C	109.5
C14—C11—C12	119.91 (13)	H22A—C22—H22C	109.5
C10—C11—C12	118.20 (13)	H22B—C22—H22C	109.5
C13—C12—C11	119.17 (12)	C8—O1—C7	118.70 (11)
C13—C12—C17	122.65 (11)	C19—O3—C20	117.07 (12)
C11—C12—C17	118.17 (12)	C21—O4—C22	116.43 (13)
